# Exacerbated Age-Related Hippocampal Alterations of Microglia Morphology, β-Amyloid and Lipofuscin Deposition and Presenilin Overexpression in *Per1*^−/−^-Mice

**DOI:** 10.3390/antiox10091330

**Published:** 2021-08-24

**Authors:** Jan Hendrik Börner, Oliver Rawashdeh, Abdelhaq Rami

**Affiliations:** 1 Institut für Experimentelle Neurobiologie (Anatomie II), Klinikum der Johann Wolfgang von Goethe-Universität, Theodor-Stern-Kai 7, 60590 Frankfurt, Germany; Hendrik-boerner@hotmial.de; 2 Chronobiology & Sleep Lab, Faculty of Medicine, School of Biomedical Sciences, The University of Queensland Brisbane, Brisbane 4072, Australia; o.rawashdeh@uq.edu.au

**Keywords:** *Per1*^−/−^-mice, aging, microglia, hippocampus, lipofuscin, autophagy, ß-amyloid, oxidative stress

## Abstract

In humans, alterations of circadian rhythms and autophagy are linked to metabolic, cardiovascular and neurological dysfunction. Autophagy constitutes a specific form of cell recycling in many eukaryotic cells. Aging is the principal risk factor for the development of neurodegenerative diseases. Thus, we assume that both the circadian clock and autophagy are indispensable to counteract aging. We have previously shown that the hippocampus of *Per1*^−/−^-mice exhibits a reduced autophagy and higher neuronal susceptibility to ischemic insults compared to wild type (WT). Therefore, we chose to study the link between aging and loss of clock gene *Per1*^−/−^-mice. Young and aged C3H- and *Per1*^−/−^-mice were used as models to analyze the hippocampal distribution of Aβ42, lipofuscin, presenilin, microglia, synaptophysin and doublecortin. We detected several changes in the hippocampus of aged *Per1*^−/−^-mice compared to their wild type littermates. Our results show significant alterations of microglia morphology, an increase in Aβ42 deposition, overexpression of presenilin, decrease in synaptophysin levels and massive accumulation of lipofuscin in the hippocampus of 24-month-old *Per1*^−/−^-mice, without alteration of adult neurogenesis. We suggest that the marked lipofuscin accumulation, Aβ42 deposition, and overexpression of presenilin-2 observed in our experiments may be some of the consequences of the slowed autophagy in the hippocampus of aged *Per1*^−/−^-mice. This may lead during aging to excessive accumulation of misfolded proteins which may, consequently, result in higher neuronal vulnerability.

## 1. Introduction

Several forms of circadian disruption are common symptoms of aging and neurodegeneration. It has also been shown that disruption of circadian rhythms accelerates the aging process [1,2]. The mammalian clock gene Period1 (*Per1*) is a central element of the circadian oscillator and has diverse extra-clock functions including cell cycle regulation, DNA damage response, and epigenetic regulation [3]. The ubiquitous expression of Per1 in many brain regions including the cerebral cortex, hippocampus and amygdala suggests that Per1 is likely also involved in other processes [3,4,5,6,7,8]. It has recently been shown that down-regulation of clock genes might initiate the development of neurological disorders [9]. Our previous in vivo and in vitro studies showed that the excessive neuronal susceptibility to ischemic insults in mice lacking Per1 (*Per1*^−/−^) is due to alterations in the expression patterns of apoptotic and autophagic markers [10,11], with higher levels of proapoptotic factors such as cytochrome c and Apaf-1 compared to wild type (WT) control mice [10]. Interestingly, the expression level of the autophagy marker LC3 was dramatically reduced in *Per1*^−/−^-mice [10,11]. Autophagy is a regulated intracellular degradation system that is involved in the degradation of cellular constituents, and management of bioenergetics sources during starvation. During autophagy, parts of the cytoplasm are removed into autophagosomes which fuse with lysosomes, thereby turning over the cytoplasmic contents [12,13,14,15,16].

Circadian defects have also been associated with age-related neurodegenerative diseases, such as Alzheimer’s disease, in which autophagy plays a primordial role in disease pathogenesis and progression [5,17]. For instance, deregulated clock genes such as *Bmal1*, which is a key component of the circadian clock in mammals, exhibit many aspects of age-related alterations [18]. Global *Bmal1*-deficient mice displayed age-associated dilated cardiomyopathy, left ventricular dilatation and contractile dysfunction [19]. In addition, *Bmal1*-mutants exhibit several alterations, such as proliferation of astrocytes, increased oxidative stress, learning and memory deficits, and altered hippocampal neurogenesis as well as a decrease in organ size and lifespan with attributes of accelerated aging [20,21,22,23].

Altered synaptic morphology, progressive loss of synapses and microglial cell activation are considered as characteristic hallmarks of aging. We assume that the slowed autophagy in the hippocampus of *Per1*^−/−^-mice during aging contributes to the accumulation of misfolded proteins and, consequently, enhances neuronal vulnerability. In the current study, we show age and genotype-dependent alterations of microglia, accumulation of lipofuscin, deposition of ß-Amyloid, and reduction in synaptophysin-levels in *Per1*^−/−^-mice.

## 2. Materials and Methods

### 2.1. Animals

All animal experiments were conducted as approved by the Policy on the Use of Animals in Neuroscience Research, the Policy on Ethics of the Society for Neuroscience, the Federal Guidelines and the European Communities Council. Mice were maintained under a standard 12/12 light/dark cycle, with 12 h light and 12 h darkness. Animals were kept under constant room temperature with food and water available ad libitum. Twenty-four adult (3 months old) and aged (24 months old) male and female C3H-mice were used (12 WT and 12 *Per1*^−/−^).

### 2.2. Immunofluorescence

Briefly, deeply anesthetized mice (chloral hydrate, 360 mg/kg body weight, Sigma, St. Louis, MO, USA) were flushed transcardially with saline, followed by perfusion with a paraformaldehyde solution (4% in 0.02 M phosphate buffered saline). Brains were post-fixed and cut into 12 μm-thick sections in the coronal plane on a freezing microtome. Sections were incubated overnight at 4 °C with primary antibodies. The following antibodies have been used: synaptophysin (Rabbit mAb, shown to exhibit sufficient specificity and selectivity for accurate quantitation of synapses; #36406 Cell Signaling, Danvers, MA, USA), doublecortin (Rabbit mAb, doublecortin is specifically expressed in neuronal precursors; Cell Signaling, #14802), LAMP1 (Rabbit mAb known as a lysosomal marker; Cell signaling, #9091), Cathepsin-B, (Rabbit mAb, Cell Signaling #31718), Iba1 (Iba1 is a protein highly expressed in microglia and macrophage with a molecular weight of about 16.7 kDa, which is a commonly known microglial marker in the nervous system; Ref. 19-19741, WAKO, Osaka, Japan), presenilin (Rabbit from Abcam, Cambridge, UK, ab51249), ß-amyloid (Rabbit pAb from Abcam, ab10148), and cleaved caspase-3 (Rabbit mAb, Cell Signaling #9661). For double staining of ß-amyloid and microglia, we used Iba1 from the mouse (IBA, from Synaptic Systems, Göttingen, Germany; mouse monoclonal antibody No. 234 011).

Briefly, tissue slides were pre-incubated for 1 h at room temperature in PBS, 5% NGS (Sigma), and primary antibodies were applied at 4 °C for 24 h in PBS plus 5% normal goat serum. Alexa Fluor 488 and Alexa 568 goat anti-mouse secondary antibodies were used at a 1:200 dilution (Molecular Probes, Göttingen, Germany). For the assessment of non-specific immunostaining, alternating sections from each experimental group were incubated without the primary antibody. All tissue sections examined in this study were processed simultaneously so that they would be exposed to exactly the same concentrations for exactly the same periods of time. We found that brain sections from aged mice showed strong autofluorescence. In some cases, the autofluorescence was so strong that the immunofluorescence of the immunostained tissues was masked and could not be quantified. For that reason, all slices were briefly incubated with a solution of 70% ethanol and incubated for 15 min with a detergent for elimination of the autofluorescence (autofluorescence eliminator reagent, EMD, Millipore Corp., Merck KGaA, Darmstadt, Germany). After rinsing the sections with 0.1 M PBS, they were mounted in Dako fluorescent mounting medium (Dako, Hamburg, Germany). Fluorescent images were acquired using an Axio-Cam digital camera mounted on a Zeiss microscope (Carl Zeiss, Jena, Germany). To assess the microglia morphology, we have measured the microglial process length and soma-size in five ROI throughout the hippocampus from each group, by using an eyepiece graticule with a scale ruled on it and calibrated with a stage micrometer.

For semiquantitative densitometric analyses of the immunoreactions, images from whole sections were digitized with an Axiocam system (Zeiss, Jena, Germany; 1030 × 1030 pixel, 8-bit color depth), using NIH ImageJ software (Image Processing and Analysis in Java, developed by Wayne Rasband), as described previously. ROI sub-regions of the hippocampal formation were selected individually, and the relative optical density (rel. O.D.) to background staining was measured within selected areas. Subsequently, values were averaged for each animal (10–12 sections per animal).

In a first step, we analyzed the signal of each marker throughout the whole hippocampus and, in the case of regional differences, we carried out the analysis in a second step individually in the dentate gyrus, CA1 and CA3 subfields. For simplification reasons, the presentation of densitometry in all hippocampal subfields is shown only when differences between the hippocampal regions are obvious and significant, as is the case with lipofuscin and synaptophysin.

### 2.3. Quantifying Lipofuscin Accumulation in the Hippocampus

Three animals (male and female) were used from each genotype and age group to assess lipofuscin levels. Brains were fixed in 4% paraformaldehyde in phosphate buffered saline (PBS) and washed with TBS, and slides were covered using Vectashield mounting medium for fluorescence containing DAPI. Section images were acquired using the Zeiss microscope system. Images were analyzed using ImageJ software (National Institute of Mental Health, Bethesda, MD, USA). For quantification pictures were converted to 8-bit grey scale and the mean signal intensities within the hippocampus were determined using the ImageJ software. For statistical analysis ten representative slides were evaluated from each animal. Groups were compared using two-way ANOVA (main factors: age and genotype) followed by the Bonferroni test. Data are presented as mean values of fluorescence intensity.

TUNEL Staining: TUNEL (terminal deoxynucleotidyl transferase UTP-biotin nick end labeling) staining was performed using In Situ Cell Death Detection Kit (Roche, Basel, Switzerland) according to the manufacturer’s instructions. Brain sections’ cells were washed twice and permeabilized with 0.1% Triton X-100 for 4 min on ice followed by incubation with TUNEL assay for 1 h at 37 °C. Brain sections were washed with PBS twice and TUNEL-positive cells were counted in five fields of the hippocampus by fluorescence microscopy and averaged.

## 3. Results

### 3.1. Lipofuscin Accumulation in the Hippocampus of Aged Mice

**Figure 1:**
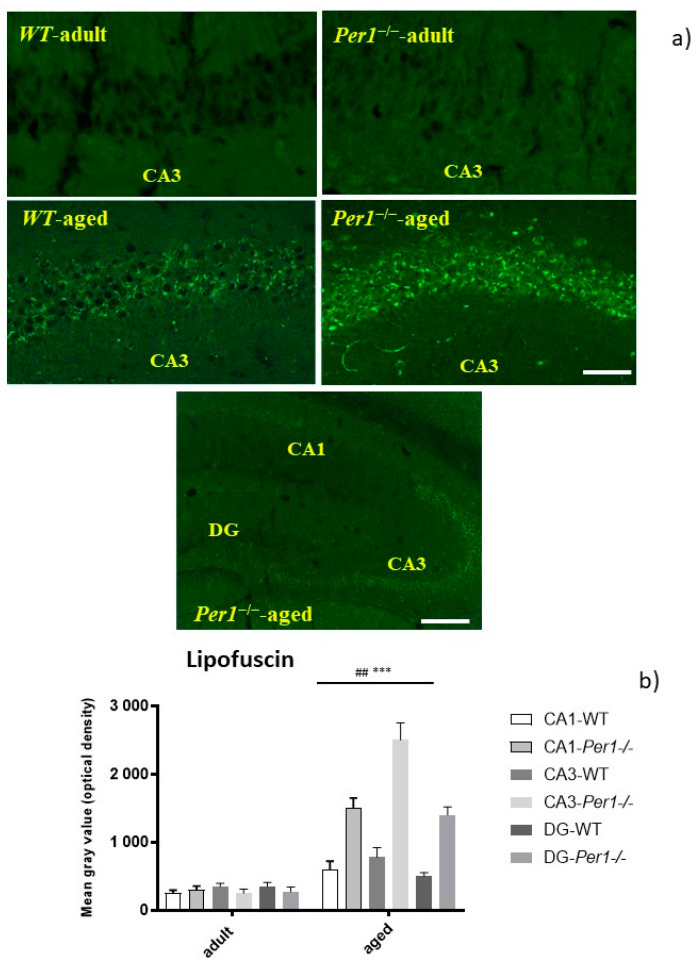
(**a**,**b**) Increased lipofuscin accumulation in the hippocampus in aged Per1^−/−^-mice. (**a**) Representative images of the CA3 region of the hippocampus in young and old wild type and Per1^−/−^-mice. Brain slices were stained with DAPI to visualize the nuclei of the cells (blue fluorescence). Lipofuscin accumulation is indicated by the presence of autofluorescence (number of slices evaluated per group = 18). Scale bar: 100 mm. (**b**) Quantification of lipofuscin autofluorescence in the hippocampus in adult and old wild type and Per1^−/−^-mice. Columns represent group mean values; error bars represent standard errors of mean (SEM). *** *p* < 0.001 difference between the age groups; ## *p* < 0.01 differences between genotypes. The formation of autofluorescent lipopigment or lipofuscin is a highly consistent and reliable cytological change that correlates with cellular aging in postmitotic cells. Autofluorescent lipofuscin in the hippocampus of aged Per1^−/−^-mice is significantly higher than in aged WT mice. Scale bar 200 µm.

We compared the intensity of lipofuscin autofluorescence in the hippocampus of young (3 months old) and aged (24 months old) WT and *Per1*^−/−^-mice. There was an age-related increase in lipofuscin fluorescence in the hippocampus. However, the accumulation of lipofuscin was significantly exacerbated in the hippocampus of *Per1*^−/−^-mice at 24 months of age, as compared to the age-matched wild type mice (Figure 1b). Interestingly, there were region-specific differences within the hippocampal formation. The lipofuscin accumulation was intensively marked in the hippocampal CA3 subfield and dentate gyrus compared to the CA1 subfield (Figure 1a) These results indicate differential age-related responses between the CA1 and CA3 subfields of the hippocampus.

### 3.2. LC3, LAMP-1 and Cathepsin-B Quantification in the Hippocampus

**Figure 2:**
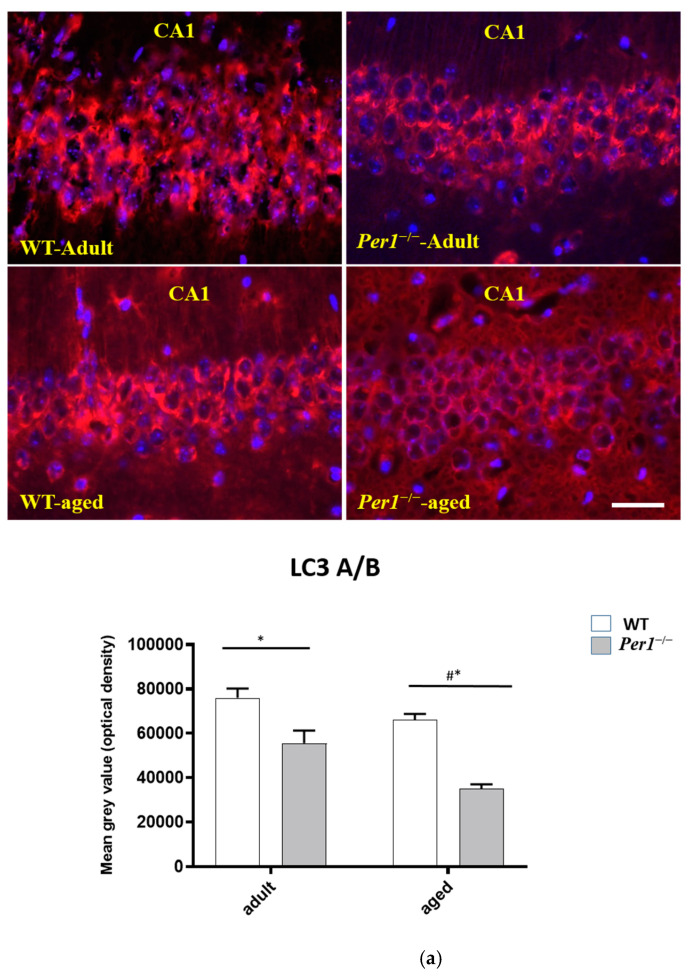

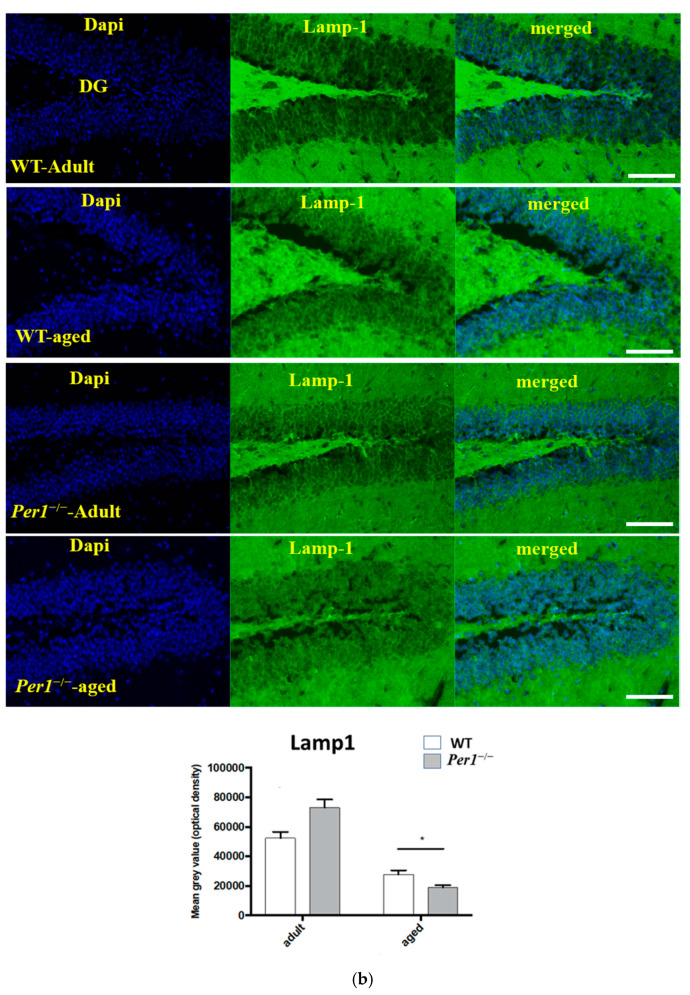

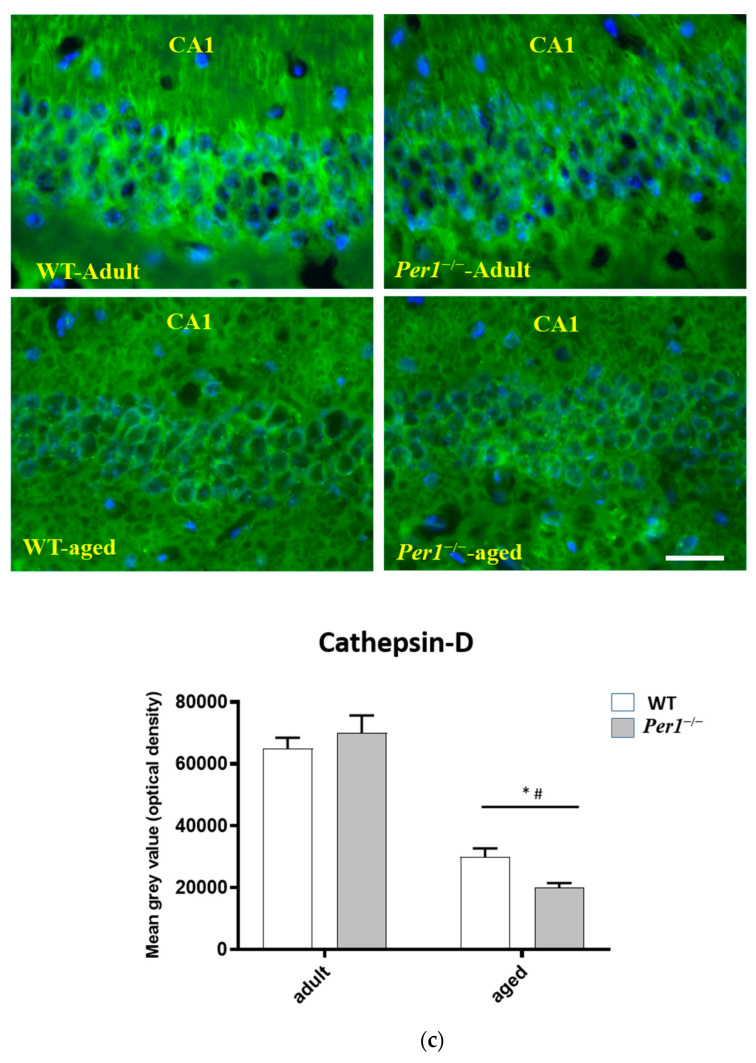
(**a**–**c**) Immunostaining and densitometric analysis of LC3A/B, LAMP1 and cathepsin-B immunoreactivities in the hippocampus of WT mice (white scale) and Per1^−/−^-mice (grey scale). The LC3 immunoreactivity was decreased in both adult and aged Per1^−/−^-mice. The LAMP-1 and cathepsin-B immunoreactivities were significantly reduced in aged Per1^−/−^-mice. Significant differences between means were statistically assessed by ANOVA. * *p* < 0.05 was considered statistically significant. * difference between the age groups; # differences between genotypes. Scale bar 200 µm.

To determine whether autophagy is altered in aged animals, we examined molecular markers of autophagy. Microtubule-associated protein 1 light chain 3 (LC3) is the mammalian homologue of yeast Atg8 that undergoes conjugation to phosphatidylethanolamine upon autophagy induction. The unconjugated LC3 (LC3-A) is localized to cytosol, whereas lipid-conjugated form (LC3-B) resides on autophagosome membrane. The total expression of LC3A/B was dramatically reduced in *Per1*^–/–^-mice, and this reduction was exacerbated in aged animals (Figure 2a).

Despite widespread distribution of LAMP1 and the heterogeneous nature of LAMP1-labeled compartments, LAMP1 is routinely used as a lysosomal marker, and LAMP1-positive structures are often referred to as lysosomes. LAMP1 is a highly N-glycosylated lysosomal membrane protein. Incomplete lysosomal degradation of lipids and proteins can lead to exacerbation of lipofuscin accumulation. In this context, we evaluated the expression of lysosomal marker LAMP-1 in the hippocampus of adult and aged WT and *Per1*^−/−^-mice. We showed that the expression of LAMP1, a major constituent of the lysosomal membrane, was significantly decreased in the hippocampus of aged *Per1*^−/−^-mice. We also evaluated the levels of cathepsin B, which is localized to the endosomal/lysosomal compartment and is primarily involved in turnover of intracellular and extracellular proteins. The levels of cathepsin-B were also significantly reduced in the hippocampus of aged *Per1*^−/−^-mice (Figure 2c).

The reduction in LAMP-1 and cathepsin B levels could explain partly the marked increase in lipofuscin accumulation in the hippocampus of aged *Per1*^−/−^-mice (Figure 2b).

### 3.3. Aging-Related Morphological Changes of Microglia in the Hippocampus

**Figure 3:**
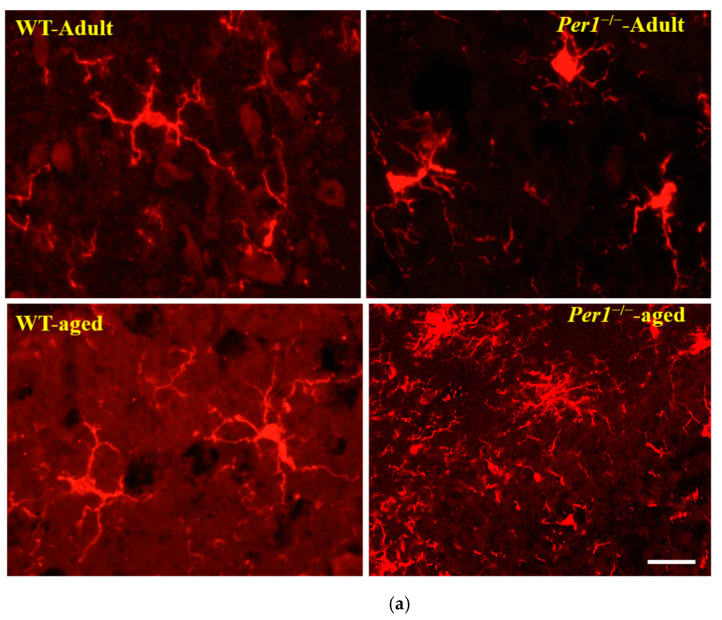

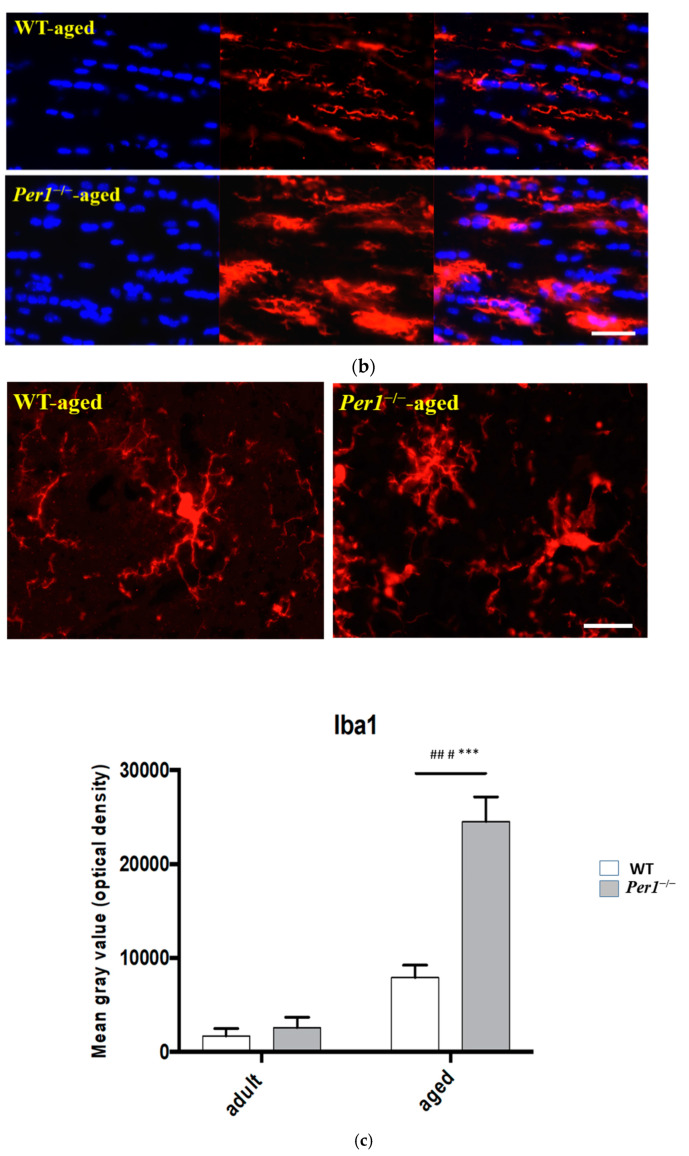

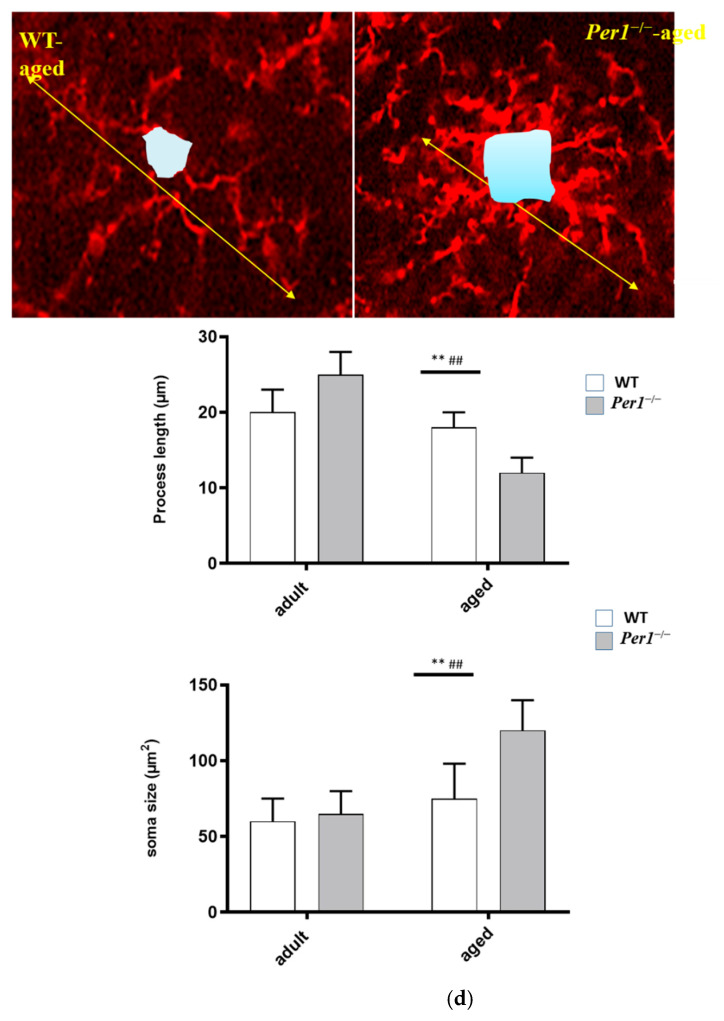
(**a**–**d**): (**a**) A homogenous distribution of hippocampal microglial cells is lost in the aged brain. Histological sections were taken from adult WT (3 months), adult *Per1*^−/−^-mice (3 months), aged WT (24 months) and aged *Per1*^−/−^-mice (24 months). Representative Iba-1 immunostained sections are shown for each age group. The comparison of clustered microglia across age groups revealed significant differences in their clustering pattern, with an increased number of microglia in close proximity to each other in aged mice. Scale bars 100 µm. (**b**) Histological sections were taken from aged WT (24 months) and aged *Per1*^−/−^-mice (24 months). The comparison of clustered microglia in the corpus callosum revealed similar differences in their clustering pattern, with an increased number of microglia in close proximity to each other in aged *Per1*^−/−^-mice. Scale bar 100 µm. (**c**) Densitometric analysis of Iba1 immunoreactivity in the hippocampus of WT (white scale) and *Per1*^−/−^-mice (grey scale). *** *p* < 0.001 and ### *p* < 0.001. (**d**) Aged *Per1*^−/−^-mice show significant alterations of microglia morphology, including soma volume increase and shortening of processes. Significant differences between means were statistically assessed by ANOVA. ** *p* < 0.01 and ## *p* < 0.01 were considered statistically significant. * Difference between the age groups; # differences between genotypes.

The microglial response in WT and *Per1*^−/−^-mice during aging was analyzed by immunohistochemistry for Iba-1 (a constitutive identity marker for monocyte-macrophage cells). A homogenous distribution of hippocampal microglial cells is lost in the aged brain. The comparison of clustered microglia across age groups revealed significant differences in their clustering pattern, with an increased number of microglia in close proximity to each other in aged mice. Microglial cells from young mice exhibit a small cell body and long slender ramifications. In contrast, microglial cells of aged mice have enlarged cell bodies and shorter, thicker processes (Figure 3a,d). These age-related alterations in microglia morphology were very pronounced in the hippocampus of aged *Per1*^−/−^-mice compared to aged WT mice. In addition, the comparison of clustered microglia in the corpus callosum also revealed similar differences in their clustering pattern, with an increased number of microglia in close proximity to each other in *Per1*^−/−^-mice (Figure 3b). In a second step, we distinguished whether changes in Iba-1 immunoreactivity resulted from a higher number of Iba-1-positive cells or from changes in Iba-1 expression at the individual cell level. The number of Iba-1-positive cells was relatively similar in both groups; however, the density of Iba-1 immunoreactivity was higher in the hippocampus of aged *Per1*^−/−^-mice compared to aged WT mice (Figure 3c).

### 3.4. β-Amyloid (Aβ42)

**Figure 4:**
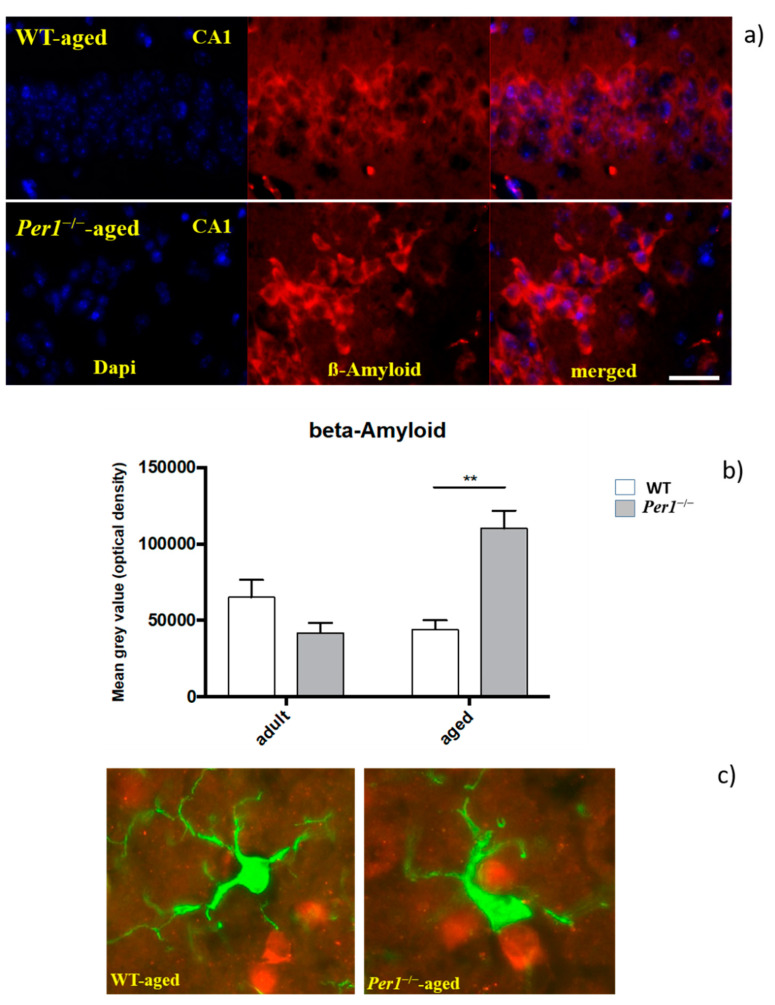
(**a**–**c**): shows an increase in Aβ42 deposition (red) in the hippocampus of aged *Per1*^−/−^-mice. Densitometric analysis of Aβ42 immunoreactivity in the hippocampus of WT (white scale) and *Per1*^−/−^-mice (grey scale). Figure 4c shows an association between microglial cells (green) and ß-amyloid (red), which is more pronounced in aged *Per1*^−/−^-mice. Microglial cell bodies (green) were located in the close vicinity of the amyloid (red). Significant differences between means were statistically assessed by ANOVA. ** *p* < 0.01 was considered statistically significant. Scale bar 100 µm in a and 50 µm in (**c**).

Immunohistochemistry was performed to determine amyloid load using an antibody directed against the N-terminal of the amyloid peptide. Quantification confirmed the age-related progression of the amyloid pathology between groups, which was pronounced in the hippocampus of *Per1*^−/−^-mice in older age (Figure 4a,b). Figure 4c shows an association of microglial cells with ß-amyloid deposits, which is more evident in aged *Per1*^−/−^-mice.

### 3.5. Presenilin-2

**Figure 5:**
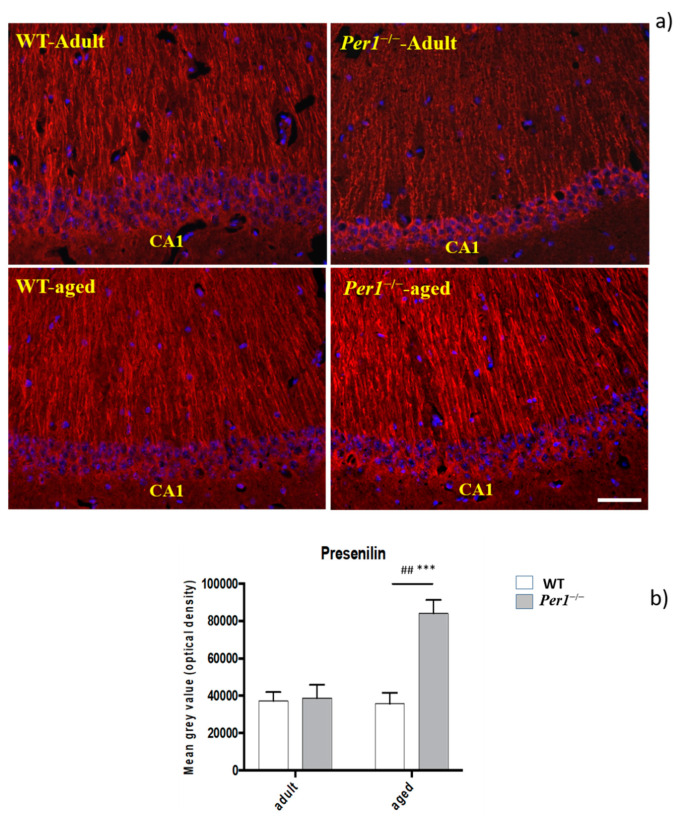
(**a**,**b**): (**a**) The presenilin immunoreactivity in the hippocampus of aged WT and aged *Per1*^−/−^-mice. (**b**) Densitometric analysis of presenilin immunoreactivity in the hippocampus of WT mice (white scale) and *Per1*^−/−^-mice (grey scale). Significant differences between means were statistically assessed by ANOVA. *** *p* < 0.001 and ## < 0.01 was considered statistically significant. Scale bar 100 µm.

Immunohistochemistry was performed to determine presenilin load using an antibody directed against presenilin-2. Quantification confirmed the age-related progression of the presenilin pathology between groups, which was pronounced in the hippocampus. Presenilin load progressed markedly in the hippocampus of *Per1*^−/−^-mice in older age (Figure 5a,b).

### 3.6. Synaptophysin Quantification in the Hippocampus

**Figure 6:**
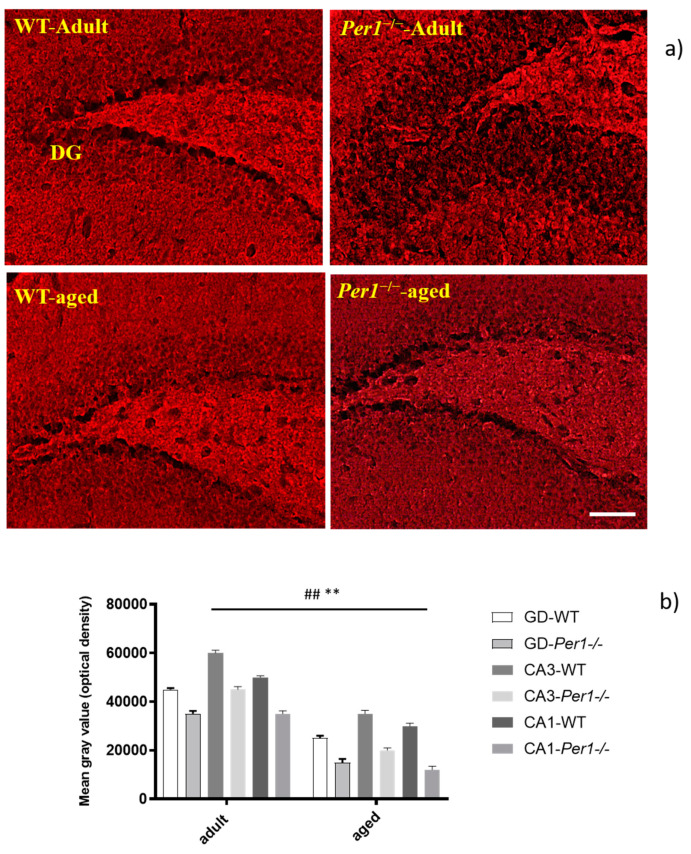
(**a**,**b**): (**a**) The synaptophysin immunoreactivity in the hippocampus of aged WT and aged *Per1*^−/−^-mice. (**b**) Densitometric analysis of synaptophysin immunoreactivity in the hippocampus of WT mice (white scale) and *Per1*^−/−^-mice (grey scale). Specific hippocampal region differences are seen when comparing dentate gyrus, CA1 and CA3 subfields. The decrease in synaptophysin levels was more pronounced in the CA3 region. Significant differences between means were statistically assessed by ANOVA. ## *p* < 0.01, ** *p* < 0.01 were considered statistically significant. Scale bar 100 µm.

Changes in numbers of specific synapses occur in development and are believed to take place in aging. An immunodetection technique to identify synaptophysin has been shown to exhibit sufficient specificity and selectivity for the accurate quantitation of synapses. In this study, we assessed semiquantitative analyses of synaptophysin immunoreactivity (IR) by optical density. Synaptophysin immunostaining was reproducible and occurred in a punctate distribution consistent with localization of presynaptic boutons. The molecular layers of dentate gyrus and other synapse dense regions of grey matter showed intense immunostaining; white-matter regions, blood vessels, and cell nuclei show negligible synaptophysin IR (Figure 6a,b). On inspection, the intensity of synaptophysin IR was significantly reduced in adult *Per1*^−/−^-mice compared to WT. Aged and adult WT mice show similar synaptophysin densities. However, aged *Per1*^−/−^-mice exhibit a dramatic reduction in synaptophysin IR compared to aged WT. The synaptophysin loss progressed markedly in the hippocampus of *Per1*^−/−^-mice in older age. Synaptophysin IR was reduced within all subfields in aged compared to young animals. Comparatively low levels of synaptophysin IR were detected in the CA1 area and dentate gyrus. These results indicate differential age-related responses between the CA1 and CA3 subfields of the hippocampus (Figure 6b).

### 3.7. Adult Neurogenesis in the Hippocampus by Quantification of Doublecortin-Positive Cells

**Figure 7:**
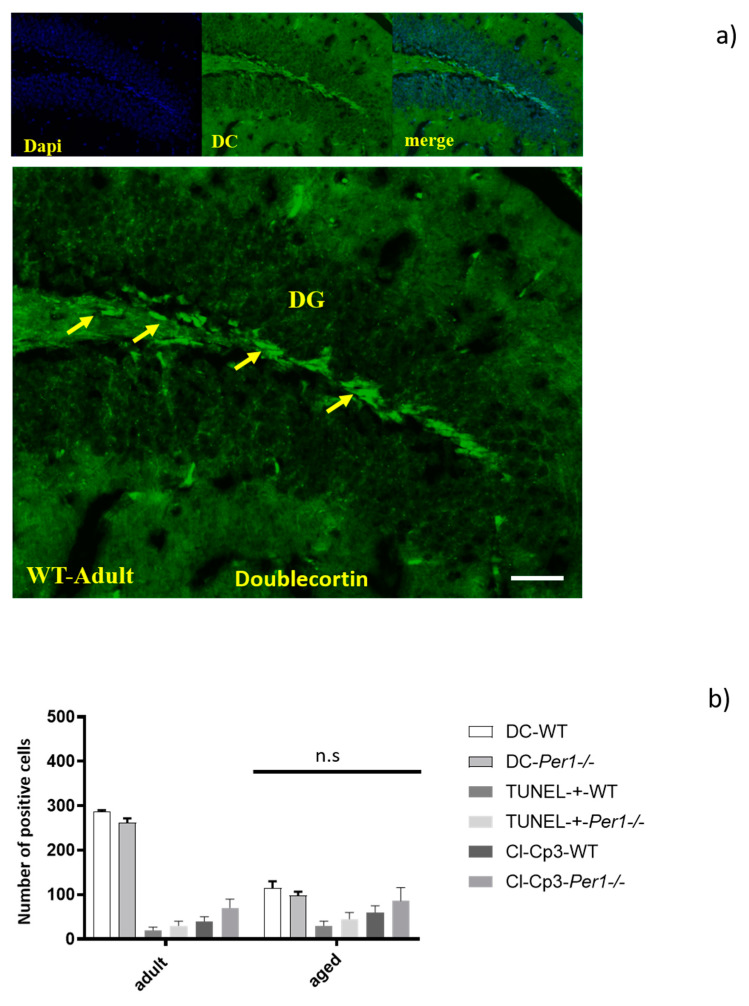
(**a**,**b**) The decreased number of Doublecortin-positive cells (DC) in the dentate gyrus was similar in both genotypes. There is a progressive decrease with age, but in a similar manner within groups (**a**). We found that Per1 deficiency did not alter the apoptotic cell death in the hippocampus in aged mice, because the density of caspase-3-positive cells as well as of TUNEL-positive cells was similar in all age groups (**b**). Therefore, we assume that Per1 has no influence on the proliferation or apoptosis of granule cells. Data are reported as means ± SD of three experiments. Scale bar 100 µm.

There is a progressive decrease in doublecortin-positive cells (DC-+ cells) with age, and the decreased number of DC-+ cells in the dentate gyrus was similar in both genotypes. The dentate gyrus of *Per1*^−/−^-mice exhibits relatively the same density of DC-+ cells. In addition, we evaluated the density of TUNEL-positive cells as well as of cleaved-caspase 3-positive cells in the hippocampus of both groups. We found that Per1 deficiency did not alter the apoptotic cell death in the hippocampus in aged mice (Figure 7b). Therefore, we can at this stage assume that Per1 has no influence on the proliferation or apoptosis of granule cells (Figure 7a,b).

## 4. Discussion

Several reports associate aging processes to malfunction of autophagy and deterioration of circadian clock functions, which influence the cellular physiology. Autophagy is in general comparable to a double-edged sword. On one hand, autophagy may help promote cell survival by generating the intracellular building blocks required to maintain vital functions during stress conditions, and on the other hand it can trigger cell death through excessive self-digestion [12,13,14,15,16]. As a matter of fact, mice lacking the autophagy genes develop symptoms of neurodegeneration [24,25,26]. In this context, we recently reported that pharmacological inhibition of autophagy using 3-methyl-adenine or bafilomycin A1 enhances susceptibility to proapoptotic signals induced by serum deprivation in hippocampal HT22 neurons [27,28,29,30,31].

The autophagic machinery declines with age, whereas its boosting induces life span extension. Genetic manipulation of autophagy-related genes improves organ function in aged rodents, and pharmacological activation of autophagy prolongs the life span of various model organisms [32]. We have previously shown that the hippocampus of adult *Per1*^−/−^-mice exhibits a defective autophagy and higher neuronal susceptibility to ischemic insults comparing to WT mice [10,29].

We hypothesized that the defective autophagy in the hippocampus of *Per1*^−/−^-mice may lead during aging to excessive accumulation of misfolded proteins, which may consequently result in higher neuronal vulnerability. Therefore, we examined the hippocampus of aged WT and aged *Per1*^−/−^-mice in terms of lipofuscin accumulation; microglia activation; Aβ42 deposition; and presenilin, synaptophysin and doublecortin expressions.

The central findings of our study are that aged *Per1*^−/−^-mice exhibit significant alterations of microglia morphology, massive accumulation of lipofuscin, an increase in Aβ42 deposition, overexpression of presenilin, and decrease in synaptophysin levels, without alteration of neurogenesis. We suggest that *Per1*^−/−^-dependent control of autophagy is altered in aged hippocampal cells and might in turn regulate the aging process.

### 4.1. Enhanced Lipofuscin Accumulation in the Hippocampus of Aged Per1^−/−^-Mice

Lipofuscin, also called “age pigment”, is a cellular waste product which accumulates during aging. Lipofuscin is known to be able to generate reactive oxygen intermediates that damage DNA [33,34]. In addition, it is known that lipofuscin can induce apoptosis through its high content in oxidized proteins [35]. In this study, exacerbated age-related lipofuscin accumulation was revealed in the hippocampus in mice lacking Per1 protein. Therefore, increased accumulation of lipofuscin selectively in the hippocampus could induce impairment in learning and memory abilities of 24-month-old *Per1*^−/−^-mice. During pathological processes, the accumulation of lipofuscin pigment would interfere with cellular functions such as the autophagic machinery and may increase probability of cell death, by increasing the accumulation of intracellular or extracellular insoluble protein aggregates in the cell. We then hypothesized that the slowed autophagy in the *Per1*^−/−^-mice might be the reason for the increased accumulation of lipofuscin. In general, lipofuscin production is due to incomplete lysosomal degradation of lipids and proteins from mitochondrial membranes. The membrane of lysosomes contains several highly glycosylated membrane proteins and lysosome-associated membrane protein (LAMPs). In addition, cathepsin B is a lysosomal cysteine protease, which mediates proteolysis within lysosomes. In general, it plays a number of roles in phagocytosis, autophagy, growth and cell proliferation. In this context, we found a significant decrease in the protein levels of lysosome-associated membrane protein 1 (LAMP-1) and cathepsin-B in aged Per1^−/−^-mice, which could potentially be responsible for lipofuscin accumulation.

### 4.2. Per1 Had No Influence on the Proliferation or Apoptosis of Granule Cells in Aged Mice

It has recently been shown that the circadian molecular clock modulates the proliferation of neural progenitor cells. For instance, time-of-day-dependent changes in proliferation were abolished in Bmal1-deficient mice [36,37]. Our study shows that hippocampal neurogenesis is reduced by age in both groups. Doublecortin-positive cells in the dentate gyrus decreased with advancing age in a similar manner in both WT and *Per1*^−/−^-mice. It is known that very few cells proliferate in the brain of rodents older than 10 months. We used doublecortin (DC) as the marker of neurogenesis in aged mice, because newborn neurons express DC during the 2 weeks after their birth, a relatively long time window that allows labelling of a relatively large number of neurogenic cells. There is a progressive decrease in DC-+ cells with age, but in a similar manner within groups. In addition, we found that *Per1* deficiency did not induce apoptosis in the hippocampus in aged mice. Therefore, we concluded that *Per1* has no influence on the proliferation or apoptosis of granule cells.

### 4.3. Enhanced Presenilin and Significant ß Accumulation in the Hippocampus of Aged Per1^−/−^-Mice

In addition, we went on to investigate whether changes in amyloid and presenilin accumulation in the aged hippocampus occurs. Indeed, there was a significant accumulation of both proteins in the hippocampus of aged *Per1*^−/−^-mice comparing to aged WT. Presenilin 2 is a transmembrane protein, initially isolated in a search for mutations associated with familial forms of Alzheimer’s disease. The major function of presenilin is its γ-secretase activity by which it proteolytically processes target proteins, including amyloid precursor protein [38], intracellular calcium regulation [39] and programmed cell death regulation [40]. Quantification of presenilin and amyloid immunoreactivities confirmed the age-related progression of the presenilin and amyloid pathologies between groups, which were more pronounced in the hippocampus of aged *Per1*^−/−^-mice.

### 4.4. Loss of Synapses and Microglial Activation in the Hippocampus of Aged Per1^−/−^-Mice

We noted an age-related reduction in the density of presynaptic marker synaptophysin within the hippocampus of *Per1*^−/−^-mice. Thus, the present results indicate that aging alters the synaptic density in the hippocampus of *Per1*^−/−^-mice. This effect may change the nature and function of the synaptic networks formed in this region and corroborate the proposal that marked regional loss of synapses precedes gross neuronal loss in aging.

Microglial cells constitute the resident macrophages of the brain [41,42]. Under physiological conditions microglial cells exhibit ramified processes, which control the surroundings. This feature allows microglial cells to reply quickly to any type of insult by altering their morphology and switching from a quiescent to a migratory state [43]. Microglial cells are able to phagocyte, clear apoptotic cells and selectively removing degenerating synapses [44]. Diminished neuroglial support at the synapse is therefore likely to alter the connectivity and, consequently, impact on cognitive capability [45,46]. Furthermore, it has been reported that age-related alterations of microglia are accompanied by an increased expression of inflammatory activation markers and changes in cytokine production. Possible consequences of these microglial alterations with aging might be a dysregulated response to injuries, changes in neuroprotective functions, and an increase in neurotoxic inflammatory responses [47]. Interestingly, the microglia morphological alterations parallel the loss of synapses within the aged hippocampus, which may suggest increased microglial–synaptic contacts.

In addition, we went on to investigate whether changes in amyloid accumulation in the aged hippocampus were associated with alterations in microglia morphology. Indeed, morphological alterations of microglial cells are accompanied by a significant Aβ accumulation. The tight association of amyloid and microglia in aged *Per1*^−/−^-mice has led to the suggestion that microglia are involved in either the phagocytosis or deposition of amyloid. However, this observation cannot unequivocally determine whether microglia are causal, contributory, or consequential to cerebral amyloidosis. Therefore, basically, the major finding is that the role of Per1 becomes more prominent with increased age. Our results predict that deletion of Per1 in the brain of aged mice could increase neuronal vulnerability to various insults.

## 5. Conclusions

Our findings show several changes in the hippocampus of aged *Per1*^−/−^-mice compared to aged WT mice. We detected significant alterations of microglia morphology, including soma volume increase and shortening of processes, an increase in Aβ42 deposition, overexpression of presenilin, decrease in synaptophysin levels and massive accumulation of lipofuscin in the hippocampus of 24-month-old *Per1*^−/−^-mice. We suggest that the increase in lipofuscin accumulation, Aβ42 deposition, and overexpression of presenilin-2 observed in our experiments may be some of the consequences of the slowed autophagy in the hippocampus of aged *Per1*^−/−^-mice. This may lead during aging to a Per1-associated excessive accumulation of misfolded proteins, which may consequently result in higher neuronal vulnerability and cognitive decline, introducing a novel function for this clock gene within neuronal homeostasis.

## Data Availability

Data is contained within the article.
